# Variability is adaptability…also during weaning!

**DOI:** 10.1186/cc13807

**Published:** 2014-03-31

**Authors:** Laurent Brochard

**Affiliations:** 1 Department of Critical Care, St. Michael’s Hospital and Keenan Research Institute, 30 Bond Street, Toronto, ON M5B 1 W8, Canada; 2 InterDepartmental Division of Critical Care Medicine, University of Toronto, Toronto, ON, Canada

## Abstract

Heart rate variability in the frequency domain can now be obtained at the bedside in the ICU. Promising data suggest that it may help to characterize a patient response to a spontaneous breathing trial. Refinement of the analysis could even help to predict the outcome of extubation or at least help to detect early patients at risk of failure. It is possible that combining this type of analysis, the breathing pattern variability, and other objective indices could help clinicians in the decision-making process of weaning and extubation.

## 

A study by Huang and colleagues [1] in the previous issue of *Critical Care* suggests that monitoring heart rate variability may be useful during weaning from mechanical ventilation. In ICUs, a spontaneous breathing trial (SBT) is today the recommended test to appreciate whether a patient under mechanical ventilation will tolerate extubation. It is an important step incorporated in the decision-making process for weaning and extubation [2]. During an SBT, the patient is placed in a situation that, for a relatively short period, best simulates what the patient will experience after extubation in terms of the work of breathing [3]. The period of this test constitutes a quasi-‘experimental’ situation during which a careful observation can help clinicians to make the best decision. The test can determine relatively well whether the patient can breathe without ventilatory support, but seems less effective at predicting what will happen after removal of the endotracheal tube. Despite careful approaches, around 15% to 20% of extubated patients will still need to be reintubated in the 72 hours after extubation. These patients have a considerably worse prognosis than those successfully extubated [4].

Huang and colleagues proposed an innovative approach to try to take advantage of the natural response of heart rate variability in situations of stress to better predict the risk of extubation failure [1]. Organ function variability seems to be a natural mechanism reflecting the adaptability of the system. In situations of stress, some elements of this variability could reflect the response of the system to constraints and the margins for adaptation. Heart rate and respiratory variability have been studied in many situations but their exact meaning is still far from being fully understood. An index derived from heart rate and from a continuous electrocardiogram analysis would be extremely appealing because this monitoring is available for every patient in the ICU. Analysis of heart rate variability can now be done in terms of frequency domain, which requires fast Fourier transform. Patients with arrhythmias need to be excluded, and the influence of diverse medications interfering with the autonomous system, the sympathovagal balance, or cardiac function is not well known. In different domains, including infections, analysis of heart rate variability has been shown to be a good predictor of the response to stress [5]. In endotoxinemia, this response is independent of fever, for instance [6]. Application of this analysis to weaning is thus attractive. In a carefully performed single-center study, the authors prospectively studied 101 patients submitted to a T-piece trial and measured classic respiratory and cardiovascular indices as well as heart rate variability: 24 patients failed their SBT, and, among the 77 who were extubated, 13 (17%) required reintubation within 72 hours. Variability in terms of very low frequency and total power spectrum were the most interesting pmeters in regard to the analysis of heart rate variability. Their data suggest that (a) a reduced variability in the frequency domain is associated with failure of SBT and (b) in extubated patients, only those with successful extubation are able to increase this heart rate variability after extubation. At the time of the SBT, however, it is not possible to differentiate patients who will ultimately fail extubation among those who tolerate the SBT. These data are promising because they indicate the possibility of an early detection of patients failing the extubation process, independently of any subjective assessment, but are still insufficient at the present time to predict the outcome of extubation. Potentially, such indices could be used to test the potential for adaptation to further stress but this would need further studies.

Breathing pattern variability has been similarly explored during weaning from mechanical ventilation, and, interestingly, compble results have already been described [7]. Variability indices were shown to be sufficient to septe success from failure cases during weaning, breathing variability being greater in patients successfully septed from the ventilator and the endotracheal tube. Complex analysis of the breathing pattern suggests that the degree of variability, of autocorrelation between breaths, or of the random fraction of variational activity indicates a different response of the breathing controller to stress and respiratory constraints [8]. During exercise, the patterns of change in heart rate variability, respiratory rate variability, and combined cardiorespiratory variability have been studied simultaneously. Measures of heart rate variability were found to be more frequently able to detect the presence of exercise, with more consistent changes across their metrics compared with breathing variables [9]. However, it is possible that, during a specific situation such as weaning from mechanical ventilation, heart rate variability and breathing pattern variability could be combined in the future to better describe the global response of the cardiorespiratory system and help the clinician in a difficult decision-making process.

## Abbreviations

SBT: Spontaneous Breathing Trial.

## Competing interests

LB’s laboratory has received research grants from Covidien (Carlsbad, CA, USA), General Electric (Fairfield, CT, USA), Vygon (Ecouen, France), and Dräger (Lübeck, Germany), and LB has been a consultant for Dräger.
